# The Relationship Between Sensory Processing and Executive Functions in Adults with and without Neurodevelopmental Disorders

**DOI:** 10.1192/j.eurpsy.2025.560

**Published:** 2025-08-26

**Authors:** S. Regev, K. Sharfi, O. Cohen Elimelech, N. Grinblat, S. Rosenblum

**Affiliations:** 1 Occupational Therapy, Ben-Gurion University of the Negev, Beer-Sheva; 2 Occupational Therapy, University of Haifa, Haifa, Israel

## Abstract

**Introduction:**

Existing literature highlights unique sensory processing patterns and decreased executive functions (EF) in adults with neurodevelopmental disorders (NDD). However, most studies have focused on specific diagnoses, such as Attention-Deficit Hyperactivity Disorder or Specific Learning Disabilities, and have used smaller sample sizes, indicating a need for broader and more comprehensive research.

**Objectives:**

Based on prior research conducted in the same laboratory, the current study aimed to comprehensively evaluate sensory processing patterns and EF in adults with NDD compared to controls, as well as to explore the relationships between these characteristics within each group.

**Methods:**

The study sample included 290 adults (aged 20–50 years), comprising 149 individuals with NDD and 141 matched controls. Participants completed the Adolescent/Adult Sensory Profile (AASP) and the Adult Behavior Rating Inventory of Executive Function (BRIEF).

**Results:**

Significant group differences were found in the AASP scores (F(4,285) = 42.05, p < .001, ηp² = 0.37), with variations in three sensory processing subscales: low registration (F(1,288) = 149.92, p < .001, ηp² = 0.34), sensitivity (F(1,288) = 103.97, p < .001, ηp² = 0.26), and avoidance (F(1,288) = 50.06, p < .001, ηp² = 0.48), though not in sensory seeking. Additionally, significant differences were observed in the BRIEF (F(5,284) = 67.58, p < .001) across all nine subscales and indexes. Notably, significant correlations were identified between BRIEF scores and three AASP subscales in both groups: low registration (control: r = .50, p < .001; NDD: r = .54, p < .001), sensitivity (control: r = .45, p < .001; NDD: r = .47, p < .001), and avoidance (control: r = .45, p < .001; NDD: r = .42, p < .001).

**Conclusions:**

This study highlights the distinct sensory processing patterns and EF challenges in adults with NDD compared to controls. The findings also reveal a consistent relationship between sensory processing and EF across both groups. These insights enhance the understanding of the interplay between sensory and executive functioning, emphasizing the importance of considering these characteristics at assessment and intervention of adults with NDD.

**Disclosure of Interest:**

None Declared

